# Efficacy and Safety of Stylage XL Lidocaine for the Restoration and/or Augmentation of Facial Volume: The Beauty Volume Study

**DOI:** 10.1093/asjof/ojad056

**Published:** 2023-06-26

**Authors:** Ali Mojallal

## Abstract

**Background:**

Hyaluronic acid (HA)-based gel fillers help correct facial volume deficits through their volumizing effect.

**Objectives:**

This postmarket clinical follow-up study was a single-center prospective cohort study designed to evaluate the efficacy and safety of Stylage XL Lidocaine (Laboratoires VIVACY, Paris, France) for the augmentation and/or restoration of facial volume.

**Methods:**

Healthy patients aged between 30 and 65 years with a facial volume grade of 3 to 5 according to the facial volume loss scale (FVLS) were considered eligible. Participants were injected subcutaneously in the area of the cheekbones (essential area). If necessary, patients were also injected in the chin, the temples, and the facial oval (optional areas). Outcomes were assessed at 1, 3, 6, 12, and 18 months following the initial treatment. A touch-up was possible at 1 month following the initial injection. The primary endpoint was the variation in the mean FVLS scores at M6 compared to baseline as evaluated by an independent assessor.

**Results:**

A total of 40 female patients (mean age of 52.5 years) were recruited between November 2019 and July 2021. There was a significant improvement in the mean FVLS score at 6 months compared to baseline (2.3 [0.6] vs 3.1 [0.6]; *P* < .0001). Patients were satisfied with the achieved aesthetic improvements and results were still observed at 12 and 18 months. Stylage XL Lidocaine also had a good safety profile and was well tolerated by the study cohort.

**Conclusions:**

The results of the 18-month Beauty Volume study confirmed the efficacy and safety of the Stylage XL Lidocaine HA-based gel filler in the augmentation and/or restoration of facial volumes.

**Level of Evidence: 3:**

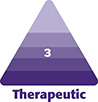

The loss of facial volume is a well-known sign of the aging process. Facial volume loss is becoming an increasingly important issue as people are not only living increasingly longer, but are also leading a more active lifestyle both socially and professionally.^1^ Hyaluronic acid (HA)-based gel fillers can help correct this volume deficit through their volumizing effect. HA-based gel fillers have been used for over 20 years for the augmentation of tissue volume. These fillers are injected below the surface of the skin to add volume, filling the lines, wrinkles, and folds, and hence, giving the skin a smoother appearance. One of the areas that suffers volume loss is the area over the cheekbones.^2,3^

Hyaluronic acid is a polysaccharide and a key molecule naturally present in the extracellular matrix with an important role in maintaining tissue structure and vascularity.^4,5^ Stylage XL (Laboratoires VIVACY, Paris, France) is a sterile HA gel, of nonanimal origin, manufactured using the patented “Inter-Penetrating Network-Like” (IPN-Like) cross-linking technology. Laboratoires VIVACY have marketed the HA-based product Stylage XL Lidocaine, a monophasic gel consisting of 1,4-butanediol diglycidyl ether (BDDE) cross-linked HA, intended for the restoration or augmentation of facial volume. This formulation includes lidocaine hydrochloride for the reduction of injection-associated pain. This postmarket clinical follow-up study was designed to evaluate the efficacy and safety of Stylage XL Lidocaine in the augmentation and/or restoration of facial volume.

## METHODS

This was a single-center prospective uncontrolled single blind study undertaken in an aesthetic center in France between November 2019 and July 2021.

### Ethics and Data Management

The study was conducted in accordance with the ethical principles outlined in the Declaration of Helsinki (1964) and the European Normalization (EN) International Organization for Standardization (ISO) (EN ISO) standard 14155:2011.^6,7^ The study also complied with Regulation (EU) 2016/679 of the European Parliament and of the Council of April 27, 2016 on the protection of persons with regard to the processing of personal data^8^ and the French Data Protection Authority law number 8-17 of January 6, 1978, modified and known as law “Informatique et Liberté” (Commission Nationale Informatique & Liberté [CNIL]). In France, this investigation was considered an interventional research study involving human beings with negligible risks and constraints according to article L1121-1 of the CSP (Category 2 research).

The clinical investigational plan and investigation documents were reviewed and received a favorable opinion by the Ethics Committee CPP ILE DE FRANCE III on August 21, 2019 (Clinical Trial Number: NCT04166292; ANSM registration number: 2019-A01404-53). Written consent was provided, through which the patients agreed to the use and analysis of their data.

### Population

Healthy patients aged between 30 and 65 years were considered to have a facial volume grade of 2 to 4 according to the facial volume loss scale (FVLS; Appendix) and wanting to have their cheekbones injected were considered eligible for the study. Potential participants had to be affiliated with a health and social security system and to agree to keep their usual cleansing/care products and apply a Sun-Protection Factor 50 cream during nonintensive sunlight exposure. Female patients with childbearing potential could only be considered if they were using hormonal or barrier contraception for at least 12 weeks prior to recruitment. Females who were pregnant, breast feeding, and planning a pregnancy were not considered eligible for the study. Furthermore, patients with a scar, mole, or anything that might affect the facial assessments, those taking immune suppressors in the previous month, or those who had retinoids within the previous 6 months were excluded from the study. Patients who had intensive sunlight or ultraviolet ray exposure were not included because the skin reaction to such rays could interfere with the device-related safety assessments. Finally, patients participating in another research study, those who have received 4500 euros indemnities for participation in research activities in the previous 12 months (including participation in the present study) were also not considered eligible for inclusion.

### Procedures and Follow-up

All potential participants were invited to a screening visit to inform them about the study and assess their eligibility for inclusion. Eligible patients who agreed to participate and provided a valid written consent were invited back and received the first injection of Stylage XL Lidocaine (Day zero, D0). All participants were injected in the cheekbones (essential area). The cheekbones injections involved injecting both the deep medial and high lateral cheek fat compartments. Injections were performed using a needle with a bolus injected in the periostea area and the rest in the deep tissues using a “fan” technique. If necessary, patients were also injected in the chin, the temples, and the facial ovals (optional areas; Video; Appendix).

Stylage XL Lidocaine contains 26 mg of HA, cross-linked with BDDE, 0.3% lidocaine hydrochloride and qs 1 mg of a phosphate buffer, and mannitol. It is available as an injectable gel in a 1 mL prefilled syringe. Participants were injected subcutaneously with 0.1 to 2 mL per treated area and up to 8 mL in total for all the facial areas on D0. Based on the patients’ and investigators’ opinions, a touch-up was possible at the first follow-up visit, 1 month after the initial treatment with a total amount of product injected limited to 4 mL for the whole face. Participants were seen for safety and efficacy assessments at 1 month (M1), where a touch up was possible, then at 3 (M3), 6 (M6), 12 (M12), and 18 (M18) months following the initial treatment. Patients were also asked to complete a daily log for the first 15 days following each injection, where they reported any injection-site reactions (ISRs).

### Study Outcomes

**Figure 1.**
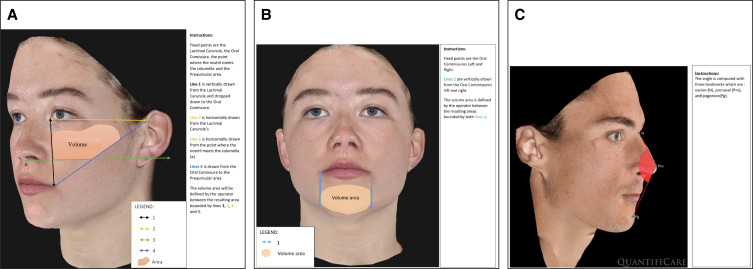
Regions of interest and instructions for (A) cheekbone and (B) chin volume quantification (shown in a 22-year-old female), and (C) chin angle calculation (shown in a 27-year-old male) on the QuantifiCare system (Biot, France).

The primary endpoint of this study was the increase in facial volume as assessed on the FVLS score at M6 compared to baseline and based on the assessment of facial photographs by an independent evaluator who was blind to the treated facial areas and the follow-up time point. Several clinical- and patient-reported outcomes were used as secondary endpoints for the study. The clinical outcomes included FVLS scores assessed on photographs by the independent assessor. The scores were used to calculate the variation in facial volume at M1, M3, M12, and M18 compared to baseline and calculate the rate of treatment responders (ie, patients with an improvement of at least 1 point on the FVLS score compared to baseline) at the same time points. Furthermore, the independent assessor also evaluated the photographs based on the global aesthetic improvement scale (GAIS) to measure the variation at each of the follow-up visits in comparison to baseline. GAIS is a 5-point Likert scale (“Very much improved,” “Much improved,” “Improved,” “No change,” or “Worse”) used to assess aesthetic improvement. A lower GAIS score indicates better improvement. For the purpose of our analysis, any of the first 3 options was considered an indication of improvement. Using a 3D QuantifiCare system (Biot, France), the variation in the cheekbones volume and, for applicable patients, the chin volume and chin angle were measured at M1, M3, M12, and M18 after initial injection (Figure 1).

With regards to patient reported outcomes, we assessed the degree of improvement, compared to prior to the injection, using the GAIS score, at M1, M3, M6, M12, and M18 and their satisfaction using the FACE-Q satisfaction with the cheekbones and, where applicable the chin questionnaires, at each of the follow-up time points. Finally, we evaluated the product tolerance by collecting information about any adverse events (AEs) or adverse device effects (ADEs) that happened to the patients throughout the study period. Additionally, ISRs were evaluated by the investigator at each study visit and by the patients themselves during the 15 days after each injection (15 days after D0 by all patients, and 15 days after the M1 visit by the patients who received touch-up injections). If an ISR was still present or appeared 15 days after the injection, and according to the investigator's discretion, the event was reported as an AE.

### Statistical Analysis

Sample size was calculated based on the assumption that the correlation coefficient between paired samples is 0.5 and that the population analyzed for the primary endpoint is not normally distributed. The sample size necessary to detect a mean difference between paired samples of −1.0 on FVLS, with an estimated standard deviation (SD) of the difference of 1.1, at a significance level and power of 5% and 90%, respectively, was estimated to be 16 patients. However, in order to adequately evaluate the safety of the device, we aimed to recruit 40 patients because this was deemed feasible.

Prior to the study, 3 analysis populations were predefined: Patients who received the tested device; those who fulfilled the inclusion/exclusion criteria and used the tested device; and those who fulfilled the study criteria without any major protocol deviations. A protocol deviation was considered major if it significantly impacted the interpretation of the study results, particularly with respect to the primary outcome. Descriptive statistics were provided for all collected data at each available time point. The mean change from baseline, along with its 95% confidence interval (CI), was calculated for the primary outcome in the population who fulfilled the inclusion/exclusion criteria, with and without replacement of missing data. Additionally, a supportive analysis was conducted on the subset population without major protocol deviations. For the secondary outcomes, changes from baseline or proportions at each time point were calculated in the population who fulfilled the inclusion/exclusion criteria without any imputation. Safety data, including AEs and local reactions, were also described.

## RESULTS

### Recruitment and Participant Characteristics

**Figure 2.**
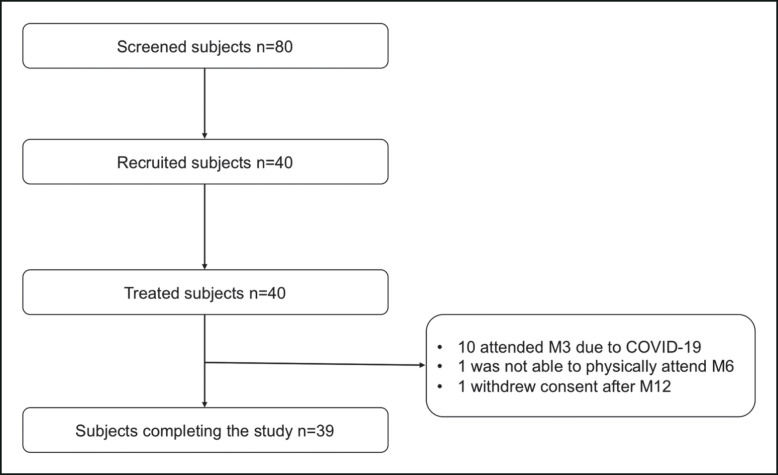
Study recruitment and follow-up flow chart.

Out of a total of 80 screened patients, 40 female patients with a mean age of 52.5 (SD 8.7; range 32-65) years and a mean BMI of 25.138 (SD 4.767; range, 17.59-39.04) were included in the study (Figure 2). A total of 19 patients had at least 1 medical history at screening visit and 13 had already received HA injection(s) ranging from 25 years to 18 months prior to the screening visit. A total of 14 patients were on medications at inclusion. The most frequently used medications were contraceptives (intrauterine contraceptive device or hormonal) and treatment for hypothyroidism. All included patients received Stylage XL Lidocaine injections in the cheekbones at D0. Additionally, 39 patients were also treated in the chin and the facial oval (the lower third of the face), and 29 were treated in the temple area. The mean volume of product injected on D0 was 3.05 (0.86) mL for cheekbones (1.57 [0.42] mL on the right side and 1.48 [0.46] mL on the left side), 1.89 (0.68) mL for the facial ovals (0.95 [0.34] mL on the right side and 0.94 [0.34] mL on the left side), 1.29 (0.46) mL for the temples (0.64 [0.23] mL on the right and left side), and 1.14 (0.45) mL in the chin. At M1, 24 (60%) patients had touch-up injections in at least 1 area. During the whole study, the quantity of Stylage XL Lidocaine injected per patient and per area varied from 0.3 to 6 mL.

Due to the travel restrictions related to the COVID-19 pandemic, only 10 participants were evaluated at the study site on M3 (for the rest, the M3 visit was performed remotely, hence, no photographs could be taken). The primary endpoint could not be assessed for 1 patient at M6 for one of the participants who was not able to attend and only had a telephone consultation because she relocated, and hence, no photographs were taken. This participant attended the M12 visit but later withdrew her consent to continue in the study and therefore did not provide data at the M18 visit (Figure 2).

### Efficacy Analysis

Based on the independent evaluator's assessment of photographs taken at baseline, 6 (15.0%), 24 (60.0%), and 10 (25.0%) had Grades 2, 3, and 4 FVLS scores, respectively. The primary endpoint was the variation in the mean FVLS scores at M6 compared to D0. The mean score was 2.3 (0.6) at M6 compared to 3.1 (0.6) at D0, and this variation was statistically significant (*P* < .0001). Furthermore, at all the other postinjection time points, the FVLS scores were statistically significantly lower than the scores obtained at baseline (*P* < .0001; Table 1).

**Table 1.** FVLS Scores, Treatment Responders, and GAIS Improvements at the Different Time Points

Variable	M1	M3^a^	M6	M12	M18
Facial volume score^IA^					
Missing data	0	30	0	0	1
Mean (SD)	−0.8 (0.9)	−0.9 (1.0)	−0.8 (0.7)	−1.2 (0.9)	−1.0 (1.0)
95% CI	−1.1; −0.5	−1.6; −0.2	−1.0; −0.6	−1.5; −0.9	−1.4; −0.7
Median	−1	−1	−1	−2	−1
Q1; Q3	−1; −1	−2; 0	−1; −1	−2; −1	−2; −1
Range	−2; 1	−2; 1	−2; 1	−2; 1	−3; 1
*P* value^w^	<.0001	<.0001	<.0001^b^	<.0001	<.0001
Treatment responders					
Missing data	0	30	1	0	1
*N* (%)	33 (82.5)	7 (70.0)	31 (79.5)	32 (80.0)	31 (79.5)
GAIS improvement^IA^					
Missing data	0	30	1	0	1
*N* (%)	40 (100)	10 (100)	38 (97.4)	39 (97.5)	39 (100)
GAIS improvement^S^					
Missing data	2	0	2	0	3
*N* (%)	36 (94.7)	39 (97.5)	37 (97.4)	35 (87.5)	31 (83.8)

Patient S565-29 had a missing value at M6. Simple imputation by mean value was applied.

FVLS, facial volume loss scale; GAIS, global aesthetic improvement scale; IA, evaluation by an independent assessor; M, month; Q, QuantifiCare volume assessment; S, Assessment by patients; SD, standard deviation; ST, Student's t; W, Wilcoxon signed-rank test.

a Due to COVID-19 restrictions 10 patients only attended the M3 follow-up and were photographed.

b Primary endpoint. Treatment responders: Patients with an improvement of at least 1 point on the FVLS score compared to baseline.

Using the GAIS scale and based on photographic evaluations made by the independent assessor, the proportion of improved patients was high over the entire duration of the study, with 100% patients improved at M1 and M3, 97.4% at M6, 97.5% at M12, and 100% at M18 (Table 1). According to the patients’ own assessments on GAIS, 94.7% and 97.5% considered themselves improved at M1 and M3, respectively. This proportion remained high at M6, with 97.4% improved patients but decreased gradually afterwards (87.5% at M12 and 83.8% at M18; Table 1).

**Figure 3.**
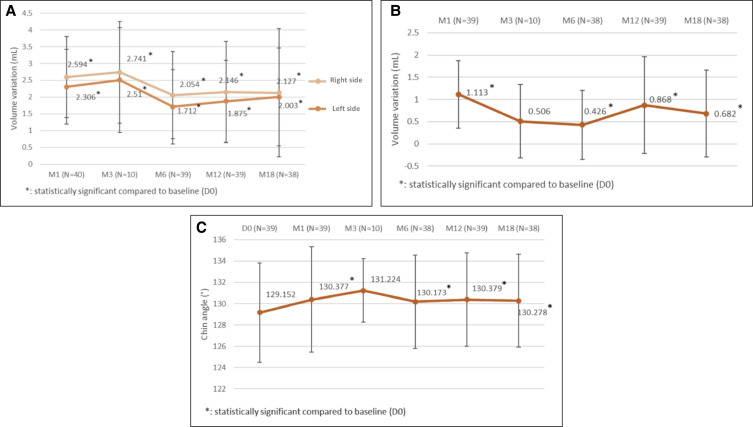
(A) Cheekbone volume, (B) chin volume, and (C) chin angle variation based on 3-dimensional (3D) acquisitions using the QuantifiCare system at each time point.

**Figure 4.**
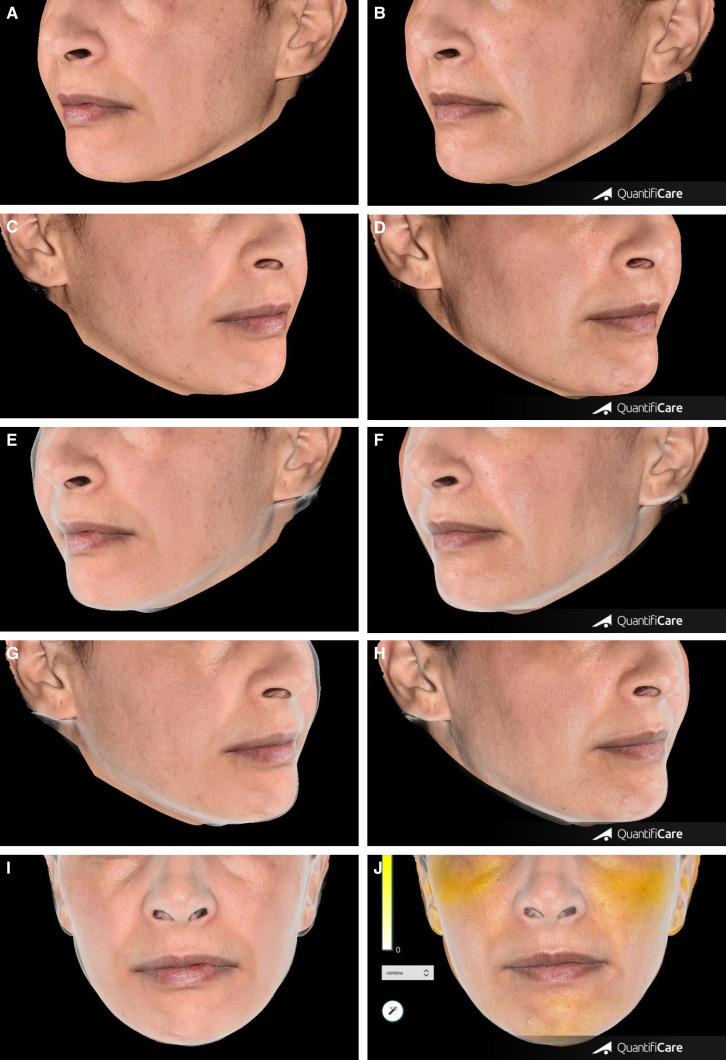
Photographs of one of the patients, a 44-year-old female, presenting with a lack of volume in the cheekbone and temple areas, chin projection and facial contouring definition. The photographs demonstrate the cheekbones’ volume changes at M6 compared to before injection: (A) left cheekbone before injection and (B) at M6; (C) right cheekbone before injection and (D) at M6; (E) left cheekbone contour silhouette before injection and (F) at M6; (G) right cheekbone contour silhouette before injection and (H) at M6; and (I) colormap front view of cheekbones before injection and (J) at M6. M, month.

**Figure 5.**
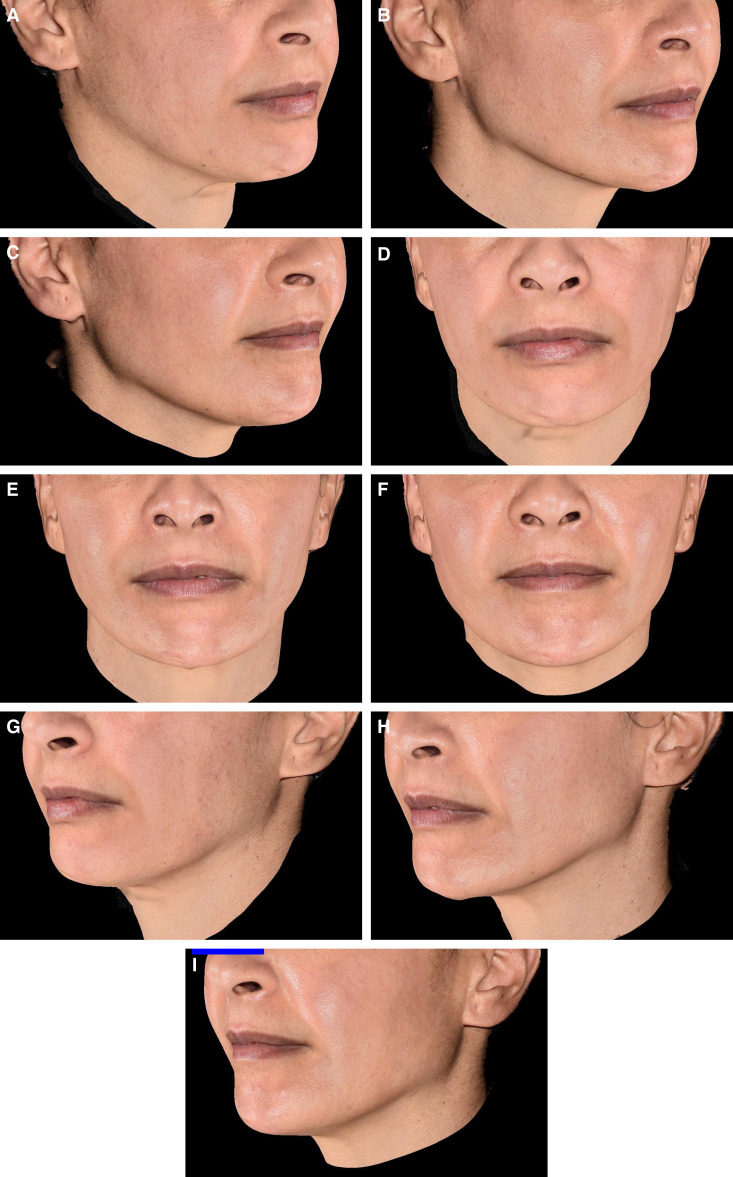
Photographs of one of the patients, a 44-year-old female, presenting with (A, D, G) lack of volume in the cheekbone and temple areas, chin projection, and facial contouring definition. The photographs demonstrate the cheekbones’ volume changes at (B, E, H) M12 and (C, F, I) M18 compared to before injection.

The variation of the cheekbone volume, chin volume, and chin angle at the different time points compared to baseline and as assessed by 3-dimensional (3D) acquisitions using the QuantifiCare system^9^ was significantly improved for all injected areas at all follow-up visits with the exception of chin volume and chin angle at M3 (Table 2, Figures 3-5). However, only 10 participants were able to be assessed at M3.

**Table 2.** Facial Volume Assessments Using the QuantifiCare System at the Different Time Points

Assessment	M1	M3^a^	M6	M12	M18
Right cheekbone volume^Q^ (*N* = 40)					
Missing data	0	30	1	1	2
Mean (SD)	2.594 (1.206)	2.741 (1.515)	2.054 (1.298)	2.146 (1.509)	2.127 (1.909)
95% CI	2.208; 2.980	1.657; 3.825	1.633; 2.474	1.657; 2.636	1.499; 2.754
Median (Q1; Q3)	2.20 (1.83; 3.14)	2.65 (1.48; 3.81)	1.61 (1.00; 3.25)	1.91 (1.17; 3.33)	1.92 (1.19; 3.28)
Range	0.45; 5.29	0.13; 5.41	0.38; 5.40	−0.54; 5.54	−2.91; 6.29
*P* value^ST^	<.0001	.0003	<.0001	<.0001	<.0001
Left cheekbone volume^Q^ (*N* = 40)					
Missing data	0	30	1	1	2
Mean (SD)	2.306 (1.117)	2.510 (1.564)	1.712 (1.108)	1.875 (1.220)	2.003 (1.460)
95% CI	1.948; 2.663	1.391; 3.629	1.353; 2.072	1.479; 2.270	1.523; 2.483
Median (Q1; Q3)	2.31 (1.38; 3.03)	1.77 (1.58; 3.89)	1.81 (0.78; 2.40)	1.82 (1.08; 2.92)	2.16 (1.05; 3.02)
Range	0.11; 4.62	0.52; 5.39	−0.70; 3.67	−0.38; 4.89	−0.72; 4.90
*P* value^ST^	<.0001	.0007	<.0001	<.0001	<.0001
Chin volume^Q^ (*N* = 39)					
Missing data	0	29	1	1	2
Mean (SD)	1.113 (0.761)	0.506 (0.827)	0.426 (0.779)	0.868 (1.089)	0.682 (0.972)
95% CI	0.867; 1.360	−0.085; 1.097	0.170; 0.682	0.511; 1.226	0.358; 1.006
Median (Q1; Q3)	1.17 (0.54; 1.56)	0.38 (0.09; 1.31)	0.53 (−0.14; 1.04)	0.83 (0.17; 1.45)	0.66 (−0.20; 1.30)
Range	−0.55; 3.19	−1.10; 1.56	−1.61; 1.83	−1.17; 4.34	−1.24; 2.52
*P* value^ST^	<.0001	.0849	.0018	<.0001	.0001
Chin angle^Q^ (*N* = 39)					
Missing data	0	29	1	0	1
Mean (SD)	1.2251 (2.9957)	0.4950 (1.0586)	0.8929 (1.7710)	1.2267 (2.0484)	0.9974 (2.0689)
95% CI	0.2540; 2.1962	−0.2623; 1.2523	0.3108; 1.4750	0.5627; 1.8907	0.3173; 1.6774
Median (Q1; Q3)	1.040 (0.360; 2.050)	0.385 (−0.230; 1.500)	1.325 (0.320; 2.060)	1.800 (0.560; 2.420)	1.590 (−0.010; 2.270)
Range	−7.380; 15.310	−1.290; 1.940	−5.880; 3.360	−7.360; 3.850	−8.470; 3.480
*P* value^ST^	<.0001	.1736	.0002	<.0001	<.0001

Patient S565-29 had a missing value at M6. Simple imputation by mean value was applied.

FVLS, facial volume loss scale; M, month; Q, QuantifiCare volume assessment; SD, standard deviation; ST, Student's t.

a Due to COVID-19 restrictions, only 10 patients attended the M3 follow-up and were photographed. Treatment responders: Patients with an improvement of at least 1 point on the FVLS score compared to baseline.

Patient satisfaction with the cheekbones and chin, for those who had a chin treatment, was assessed using the FACE-Q questionnaire. The rasch score allows the interpretation of the data obtained via the FACE-Q satisfaction with cheekbones questionnaire. Higher scores reflect a better outcome, with a maximum effect at 100. The calculated mean rasch scores at baseline for the cheekbones and chin satisfaction were 34.8 (17.2) and 37.6 (16.7), respectively. At all postinjection time points, the rasch score was higher compared to baseline, and this increase was statistically significant (*P* < .0001).

### Safety Analysis

#### Injection-Site Reaction

On D0, after the first injection and based on the investigator's evaluation, 37 (92.5%), 9 (22.5%), and 4 (10%) patients had redness, swelling/edema and pain/tenderness over the cheekbone(s), respectively, while 1 patient (2.5%) experienced bruising at the injection site. All these ISRs subsided by the M1 visit except for injection-site pain/tenderness in the cheekbone area in 1 participant. Of the participants who had a touch-up injection in at least 1 cheekbone (*n* = 18), 7 (38.9%) experienced redness, 4 (22.2%) had lumps/bumps, 2 (11.1%) reported pain/tenderness, and 1 (5.6%) had swelling/edema after the touch-up injection, according to the investigator. There were no ISRs reported by the investigator at M3, M6, or M12. However, at M18, 1 participant (2.6%) presented with an induration on the cheekbones. This event was followed up and resolved spontaneously 1 month later. All the ISRs reported by the investigator on the cheekbones were of mild intensity.

Following the initial chin injection, the investigator reported that 29 (74.4%) patients experienced redness, while, swelling/edema, pain/tenderness, and bruising at the injection site were experienced by 2 participants (5.1%) each. One of these was considered to still have some injection-site induration by the investigator at M1. At M1, 3 participants had chin touch-up injections, and the investigator reported redness, pain/tenderness, and lumps/bumps as ISRs in one of them. No chin ISRs were reported at the rest of the visits. Similar to the cheekbones, all the investigators reported chin ISRs were of mild intensity.

A total of 39 patients were injected in 1 or both facial ovals. According to the investigator, 12 (30.8%) of these patients experienced redness, 4 patients (10.3%) experienced lumps/bumps, 3 patients (7.7%) experienced pain/tenderness, and 1 patient (2.6%) experienced swelling/edema following the initial injection. Of these, 4 (10.3%) still had an ISR in the form of lumps/bumps at M1 prior to any touch-up treatments. Of the 18 participants who had touch-up injections over this area, 7 (38.9%), 6 (33%), 3 (16.7%), 2 (11.1%), and 1 (5.6%) were considered, by the investigator, to have redness, lumps/bumps, bruising, pain/tenderness, and swelling/edema, respectively. No ISRs were reported in relation to the facial ovals by the investigator at M6 and M18. However, lumps/bumps and redness were reported in 1 participant each at M3 and redness in 1 participant at M12. All the ISRs reported were considered mild except for 1 which was rated moderate in intensity.

**Figure 6.**
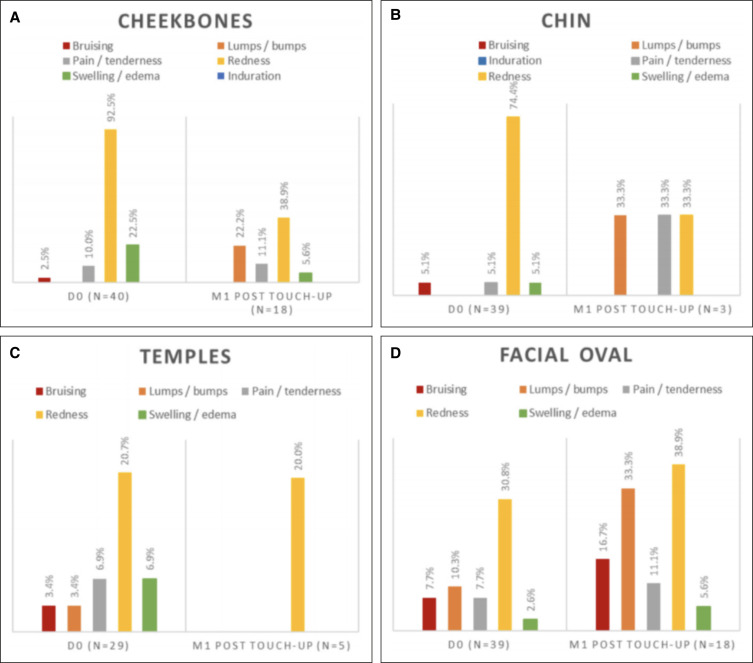
Injection-site reaction in (A) cheekbones, (B) chin, (C) temples, and (D) facial oval based on the investigator's assessments.

**Figure 7.**
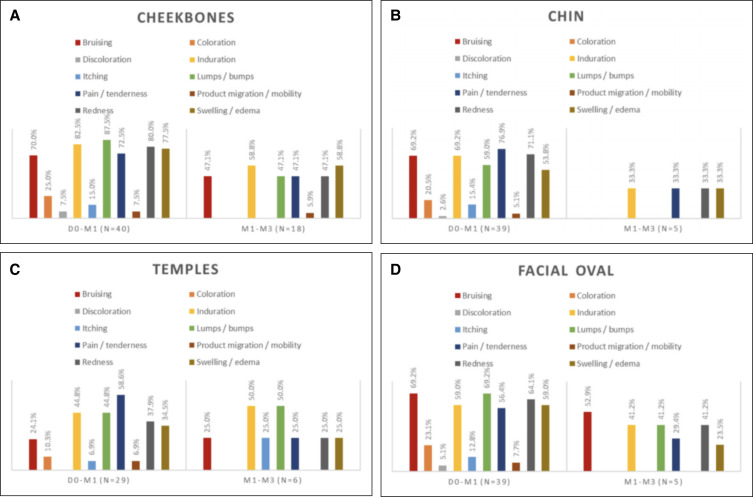
Injection-site reaction in (A) cheekbones, (B) chin, (C) temples, and (D) facial oval based on patients’ assessments.

One or both temples were injected in 29 patients on D0. Of these, the investigator reported that 6 (20.7%) had redness, 2 (6.9%) experienced pain/tenderness, 2 (6.9%) had swelling/edema, 1 (3.4%) had lumps/bumps, and 1 (3.4%) experienced bruising following the initial injection. All these subsided when reviewed at M1 before any touch-up treatments. A total of 5 patients received touch-up injections in the temples at M1 and 1 ISR (20%) in the form of redness was reported by the investigator in these patients. No ISRs were reported at the remaining follow-up visits, and all the ISRs were considered to be of mild intensity. The summary of ISRs reported by the investigator and patients (during the first 15 days following injections) is presented in Figures 6 and 7.

#### Adverse Events (AEs) or Adverse Device Effects (ADEs)

During the studied period, a total of 157 AEs were reported in 33 patients. Of these AEs, 150 were of mild intensity, 6 of moderate intensity, and 1 of severe intensity. One serious AE (SAE) was reported in 1 participant who had a dislocation of the right patella, which led to her hospitalization for 2 days. This event was not linked to the medical device. Of the 157 reported AEs, 30 were expected ADEs. The reported ADEs included swelling, induration, pain, redness, bruising and lumps, or bumps related to the injected area. Among these, only 1 event was considered to be of moderate intensity (pain in the jaw felt when opening the mouth and chewing), and the remaining ADEs were of mild intensity. All reported ADEs were expected and already described in the instructions for use of the Stylage XL Lidocaine product. All, but 7 ADEs, resolved in <30 days. The ADEs lasting up to 50 days were facial oval lump, induration on the chin and on the cheekbone, lump on the temple, redness on the facial oval, erythema on the mandibles, and bumps on the right cheekbone.

## DISCUSSION

### Summary of the Study Findings

This prospective uncontrolled postmarketing clinical investigation was designed to assess the efficacy and safety of Stylage XL Lidocaine for the restoration and/or augmentation of facial volumes, over an 18-month follow-up period. Healthy patients aged between 30 and 65 years, with a clinically evaluated facial volume deficiency (FVLS Grades 3-5), requiring treatment in, at least, the cheekbone area and willing to undergo treatment with HA dermal fillers were recruited at 1 investigational center in France. The primary outcome was assessed on FVLS by an independent evaluator blind to the treated area using 3D photographs. The mean decrease in the FVLS score from baseline was −0.8 (0.7) at 6 months (*P* < .0001), indicating an improvement (ie, increase) in the facial volumes. Furthermore, mean FVLS scores were significantly reduced at all the other follow-up time points, as evaluated by the independent assessor. The improvements in the treated areas were also confirmed both by the independent assessor and the participants themselves on the GAIS scale and by the participants’ satisfaction on the FACE-Q questionnaire. The safety of Stylage XL Lidocaine was assessed by the evaluation of ISRs (by the investigator and by the patients themselves) and by the standard reporting of AEs and ADEs. All reported events were expected and were already included in the product instructions for use.

### Results in Light of What is Already Known

Treatment responders, defined as patients with an improvement of at least 1 point on the FVLS compared to baseline, were high at all time points. Previous studies on cross-linked HA fillers, that used the FVLS, reported 100% treatment responders for up to 3 months after the initial injection(s).^10,11^ In these studies, the scales were evaluated by the investigators. In the present study, the facial volumes were independently evaluated by a single assessor on photographs using FVLS. The assessor was blind to the treated area(s) and the assessment time point. The 2 aforementioned studies also showed a decrease in the rates of treatment responders over time down to 57% at 6 months^11^ and 61% at 9 months.^10^ Our findings indicate that the volumizing effect of Stylage XL Lidocaine is likely to be more stable over time, with a treatment responder rate of 79.5% reported 18 months after the initial injections. The sustainability of the volumizing effect of Stylage XL Lidocaine was also objectively confirmed using 3D image quantification. The long-term improvement of facial contour and patient satisfaction with Stylage XL for the management of HIV-related lipoatrophy was previously demonstrated. Nonetheless, the follow-up duration and sample size of the Beauty Volume study are longer and larger, respectively.^12^ Similarly, Cohen et al reported on the efficacy and safety of Stylage XL for vectorial facial sculpting in a case series of 45 patients. However, the duration of the follow-up was 3 months.^13^ These 2 previous studies used Stylage XL preparations without lidocaine.

In previous studies evaluating the aesthetic effects of cross-linked HA using the GAIS, the decrease in the proportion of aesthetically improved patients was more marked in the patients’ self-assessments compared to those of the independent evaluators. Indeed, in a split-face study, the blinded evaluator rated 100% of patients as improved on both sides of the face at 4, 12, and 24 weeks. While, according to the participants’ evaluations, although all participants considered that both sides of the face were improved at 4 weeks, 96.39% only considered themselves improved on both sides by 24 weeks.^14^ Similarly, Beer et al reported that 94.0% of their patients were “improved” at 1 month and 91.2% at 6 and 12 months, based on the investigator's assessments. However, based on participants, 87.3% and 82.4% only considered themselves improved at 6 and 12 months, respectively.^15^ In the present study, the proportion of “aesthetically improved” patients remained high (97.4%) 6 months after the initial injections based on both the independent reviewer's evaluation and patients’ self-assessments. Nonetheless, the declining rates of improvement at the subsequent time points suggest that the aesthetic efficacy of Stylage XL Lidocaine wanes over time.

An increase in the chin angle indicates better aesthetic characteristics and more balanced global facial features, as it creates harmony in the patient's profile and the nose–lips–chin ratio.^15,16^ In this study, the chin angle (top of the nose–tip of the nose–pogonion) was calculated from the 3D acquisitions performed at baseline and at all subsequent time points (Figure 1). Following the Stylage XL Lidocaine chin injections, with the exception of M3, there was a statistically significant increase in the chin angle compared to baseline. These changes support the global aesthetic improvements of the filler. To our knowledge, very few studies have considered the change in the chin angle as a criterion for the evaluation of the performance of HA fillers. Of the few studies evaluating this parameter, the 1 performed by Beer et al measured the glabella–subnasal–pogonion angle before and 12 months after chin injection and found an increase of 1.28° in the chin angle, which is consistent with the results obtained in the Beauty Volume study.^15^

### Strengths and Limitations

Our study has several strengths, which increase the internal validity and generalizability of our findings. First, the volumizing effect of Stylage XL Lidocaine was objectively confirmed by 3D image quantifications. The 3D image quantification system developed by LifeViz (QuantifiCare) was used to detect variations in the volumes of each of the cheekbones and the chin at the different follow-up time points compared to baseline. The LifeViz 3D system used in this study is composed of a stereo-vision digital camera equipped with dual beam pointers, ensuring the high quality and reproducibility of the pictures, hence favoring accurate comparisons. Furthermore, the investigational staff ensured that all the photographs were taken in the same room with similar lighting conditions and participants’ positions to enable the detection of subtle volume variations and mitigate any systematic bias. Second, all the injections were performed by 1 operator using the same technique, which mitigated the risk of heterogeneity that could have been introduced by interoperator variability. Third, the primary outcome was based on evaluations by an expert assessor, independent of the study center and sponsor, who was blind to the treatment area(s) and the follow-up time point. Moreover, we reported a core set of clinical and patient reported outcomes using validated measures that are commonly reported in aesthetic facial studies to facilitate data comparison and data pooling with other reports. Finally, the prospective recruitment of the target sample size gives us confidence that our study was appropriately powered to detect a true difference in our selected outcomes. However, we also appreciate that the low number of patients who were able to attend the planned follow-up visit 3 months after the injection is a limitation. This was as a result of the COVID-19 imposed travel restrictions and hence beyond our control. Nevertheless, this did not affect the primary outcome of the study and was, indeed, followed by several other follow-up time points that had optimal follow-up rates. Furthermore, the lack of a control arm in our study could be perceived as a limitation to our study design. However, our study design ensured that each participant acted as their own control. Finally, the use of 1 assessor was a limitation to this study; nevertheless, the evaluator's assessments were objectively confirmed by 3D image quantification.

## CONCLUSIONS

The results of the 18-month Beauty Volume study confirmed the efficacy of the Stylage XL Lidocaine dermal filler in the augmentation and/or restoration of facial volumes. Patients were satisfied with the aesthetic improvements achieved by the treatment. Moreover, the response to treatment and the rate of treatment responders were high based on independent and objective evaluations. However, there was a slight and gradual decrease in these improvements overtime, which is an expected outcome with resorbable HA fillers. The study also demonstrated that Stylage XL Lidocaine has a good safety profile and was well tolerated by the study cohort. Indeed, all the ISRs and reported adverse device events fell within the remit of expected side-effects and are already described in the current instructions for use of the product. To further support the results of this study, it would be interesting to conduct a comparative study, assessing the efficacy and safety of Stylage XL Lidocaine to those of other marketed HA fillers with a similar composition.

## Supplementary Material

ojad056_Supplementary_Data
