# Supplementary material for: Efficacy and Safety of Stylage XL Lidocaine for the Restoration and/or Augmentation of Facial Volume: The Beauty Volume Study
Source: Aesthet Surg J Open Forum. 2023 Jun 26;5:ojad056. doi: 10.1093/asjof/ojad056 (PMC10494779; doi:10.1093/asjof/ojad056)
Supplement: ojad056_Supplementary_Data [file ojad056_Supplementary_Data.zip › 23-0025_Appendix.docx]

**Appendix:** The Beauty Volume Study
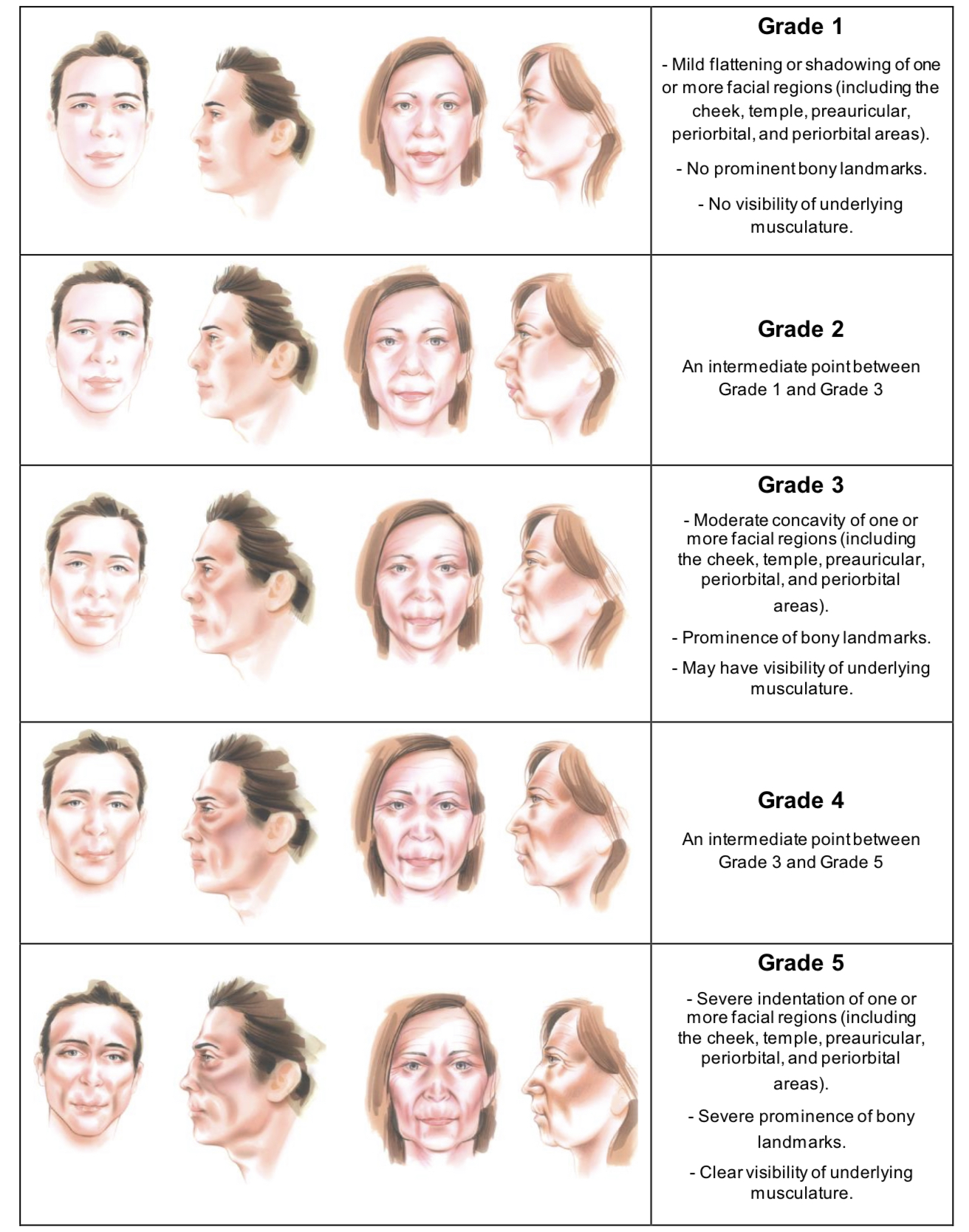
 - The Facial Volume Loss Scale (FVLS)

CHEEKBONES:

3 tracings are made: 1^st^ on the palpebral jugal junction, 2^nd^ at the level of the midcheek groove and 3^rd^ vertical line at the level of the lateral canthus.

3 boluses are performed: a first bolus is performed at the intersection between the 2nd and 3rd lines. A second bolus is performed supra-laterally along the zygomatic bone. A third bolus located laterally to the infraorbital foramen is injected supraperiosteally.

The injections are performed with a 27G needle.

CHIN:

2 tracings are made : a 1^st^ vertical line of the median axis of the face, a 2^nd^ horizontal line on the edge of the mandibular symphysis.

One bolus is realized perpendicular to the intersection of these two lines in the central area of the chin. Then from the same entry point, we inject to the left and right along the basilar edge in a retrograde linear injection.

The injections are performed with a 27G needle.

TEMPLE:

The upper border of the zygomatic arch, the lateral border of the orbital frame and the temporal crest are drawn. The position of the sentinel vein and the frontal branch of the superficial temporal artery are marked.

A first injection is carried out perpendicularly in the upper half of the temporal region, using the gunshot technique. This injection is carried out perpendicularly, crossing the temporal facies and going towards the temporal fossa to carry out a bolus under the deep temporal facies. This injection is performed with a 27G needle.

Then, a second injection, with a 25G cannula via an entry point on the temporal ridge. This single point of entry allows the entire temporal region to be injected in a retrograde fan (retro tracing) under the superficial facia temporalis.

FACIAL OVAL:

To define the lower oval of the face, a harmonious line from the mandibular angles to the chin is created. For this, a first injection with a 27G needle perpendicular to the skin and seeking the bone contact is carried out at the mandibular angle in a deep bolus. From this entry point, a fan-shaped needle injection in the subcutaneous plane is performed to fill the entire triangle representing the mandibular angle. Then a vertical linear injection is performed at this level constituting a structural column.
